# Dichlorido(2,9-dimethyl-1,10-phenanthroline)manganese(II) hemihydrate

**DOI:** 10.1107/S1600536809049149

**Published:** 2009-11-21

**Authors:** Xinfang Wang

**Affiliations:** a Department of Chemistry, Dezhou University, Dezhou 253023, People’s Republic of China

## Abstract

In the title compound, [MnCl_2_(C_14_H_12_N_2_)]·0.5H_2_O, all of the non-H atoms apart from the Cl atom lie on a mirror plane and the methyl H atoms are disordered over two sites of equal occupancy about the mirror plane. The Mn^II^ ion is coordinated in a distorted tetra­hedral environment by two N atoms of the phenanthroline ligand and two chloride ions. A half-occupancy solvent water mol­ecule lies on a mirror plane and close to an inversion center.

## Related literature

For related crystal structures, see: McCann *et al.* (1998 ▶); Pan & Xu (2005 ▶); Xu *et al.* (2009 ▶).
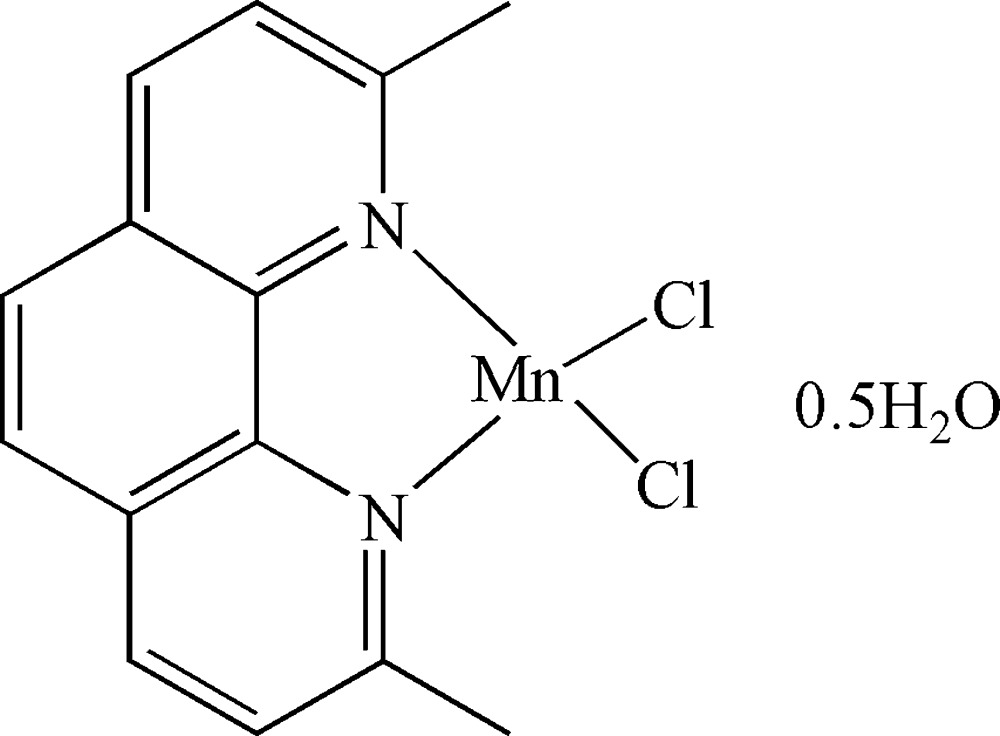


## Experimental

### 

#### Crystal data

[MnCl_2_(C_14_H_12_N_2_)]·0.5H_2_O
*M* *_r_* = 343.10
Monoclinic, 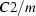

*a* = 18.763 (4) Å
*b* = 7.7343 (15) Å
*c* = 11.362 (2) Å
β = 101.532 (3)°
*V* = 1615.5 (5) Å^3^
*Z* = 4
Mo *K*α radiation
μ = 1.14 mm^−1^
*T* = 293 K
0.31 × 0.23 × 0.19 mm

#### Data collection

Bruker APEXII CCD area-detector diffractometer
Absorption correction: multi-scan (*SADABS*; Sheldrick, 2008*a* ▶) *T*_min_ = 0.719, *T*_max_ = 0.813
4794 measured reflections
1511 independent reflections
1199 reflections with *I* > 2σ(*I*)
*R*_int_ = 0.020

#### Refinement

*R*[*F* ^2^ > 2σ(*F* ^2^)] = 0.044
*wR*(*F* ^2^) = 0.134
*S* = 1.01
1511 reflections
120 parameters
.
Δρ_max_ = 0.59 e Å^−3^
Δρ_min_ = −0.31 e Å^−3^

### 

Data collection: *APEX2* (Bruker, 2004 ▶); cell refinement: *SAINT-Plus* (Bruker, 2001 ▶); data reduction: *SAINT-Plus*; program(s) used to solve structure: *SHELXS97* (Sheldrick, 2008*b* ▶); program(s) used to refine structure: *SHELXL97* (Sheldrick, 2008*b* ▶); molecular graphics: *SHELXTL* (Sheldrick, 2008*b* ▶); software used to prepare material for publication: *SHELXTL*.

## Supplementary Material

Crystal structure: contains datablocks I, global. DOI: 10.1107/S1600536809049149/lh2953sup1.cif

Structure factors: contains datablocks I. DOI: 10.1107/S1600536809049149/lh2953Isup2.hkl

Additional supplementary materials:  crystallographic information; 3D view; checkCIF report

## Figures and Tables

**Fig. 1.**
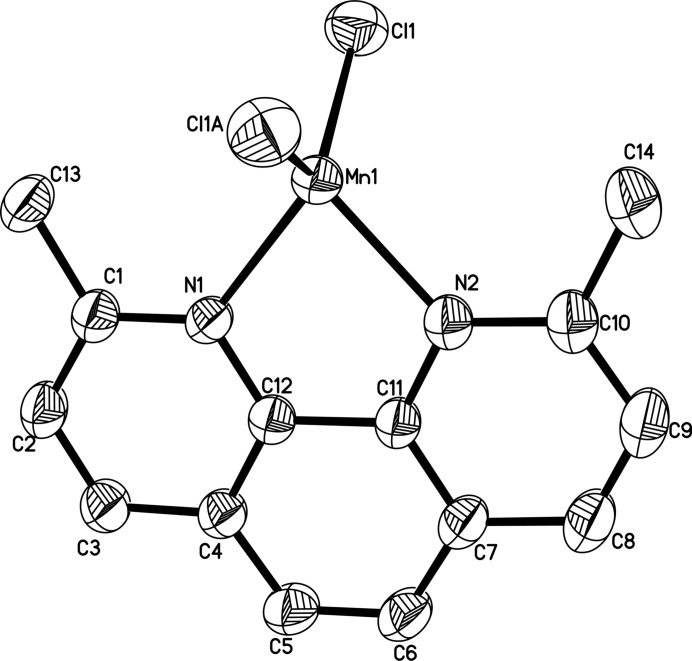
The molecular structure of the title compound with the atom-labelling scheme. Displacement ellipsoids are drawn at the 30% probability level. The disordered water molecule and all the H-atoms are omitted for clarity.

## supplementary crystallographic information

### Comment

1,10-phenanthroline is a good bidentate chelating ligand and here, we present the crystal structure of the title complex based on 2,9-dimethyl-1,10-phenanthroline.

The crystal structure of the title compound is shown in Fig. 1. The coordination environment of the Mn^II^ ion is distorted tetrahedral, in which two sites are occupied by the two N atoms of the chelating 2,9-dimethyl-1,10-phenanthroline ligand and the other two from two chloride ions. For Mn—N and Mn—Cl bond lengths in other manganese biphenanthroline complexes, see e.g. McCann, *et al.* (1998); Pan & Xu (2005); Xu *et al.* (2009). The location of the water H atoms is such that they are disordered over several sites imposed by the crystal symmetry and hence any potential hydrogen bonding is not discussed.

### Experimental

A mixture of 2,9-dimethyl-1,10-phenanthroline, MnCl_2_.4H_2_O (1:2, molar ratio) and water (20 ml) was sealed in a Teflon-lined autoclave (25 ml) and heated 393 K for two days. Upon cooling slowly and opening the bomb, yellow crystals suitable for X-ray diffraction were obtained with a yield about 40% (based on phenanthroline).

### Refinement

All H atoms bonded to C atoms were included using the HFIX commands in SHELXL-97 (Sheldrick, 2008b/i>) with C—H distances of 0.93 and 0.96 Å, and were allowed for as riding atoms with U_iso_(H) = 1.2U_eq_(C) and 1.5U_eq_(C_methyl_). The H atoms of the disordered water molecule were found in a difference Fourier map and were refined as riding with O-H fixed at 0.85 Å and U_iso_(H) = 1.2U_eq_(O).

### Crystal data

**Table:**

[MnCl_2_(C_14_H_12_N_2_)]·0.5H_2_O	*F*(000) = 696
*M**_r_* = 343.10	*D*_x_ = 1.411 Mg m^−^^3^
Monoclinic, *C*2/*m*	Mo *K*α radiation, λ = 0.71073 Å
Hall symbol: -C 2y	Cell parameters from 1089 reflections
*a* = 18.763 (4) Å	θ = 2.9–26.8°
*b* = 7.7343 (15) Å	µ = 1.14 mm^−^^1^
*c* = 11.362 (2) Å	*T* = 293 K
β = 101.532 (3)°	Block, yellow
*V* = 1615.5 (5) Å^3^	0.31 × 0.23 × 0.19 mm
*Z* = 4	

### Data collection

**Table:**

Bruker APEXII CCD area-detector diffractometer	1511 independent reflections
Radiation source: fine-focus sealed tube	1199 reflections with *I* > 2σ(*I*)
graphite	*R*_int_ = 0.020
φ and ω scans	θ_max_ = 25.0°, θ_min_ = 2.2°
Absorption correction: multi-scan (*SADABS*; Sheldrick, 2008*a*)	*h* = −22→22
*T*_min_ = 0.719, *T*_max_ = 0.813	*k* = −8→9
4794 measured reflections	*l* = −13→13

### Refinement

**Table:**

Refinement on *F*^2^	Primary atom site location: structure-invariant direct methods
Least-squares matrix: full	Secondary atom site location: difference Fourier map
*R*[*F*^2^ > 2σ(*F*^2^)] = 0.044	Hydrogen site location: inferred from neighbouring sites
*wR*(*F*^2^) = 0.134	*w* = 1/[σ^2^(*F*_o_^2^) + (0.1015*P*)^2^] where *P* = (*F*_o_^2^ + 2*F*_c_^2^)/3
*S* = 1.01	(Δ/σ)_max_ = 0.001
1511 reflections	Δρ_max_ = 0.59 e Å^−^^3^
120 parameters	Δρ_min_ = −0.31 e Å^−^^3^
0 restraints	

### Special details

**Table:**

Geometry. All e.s.d.'s (except the e.s.d. in the dihedral angle between two l.s. planes) are estimated using the full covariance matrix. The cell e.s.d.'s are taken into account individually in the estimation of e.s.d.'s in distances, angles and torsion angles; correlations between e.s.d.'s in cell parameters are only used when they are defined by crystal symmetry. An approximate (isotropic) treatment of cell e.s.d.'s is used for estimating e.s.d.'s involving l.s. planes.
Refinement. Refinement of *F*^2^ against ALL reflections. The weighted *R*-factor *wR* and goodness of fit *S* are based on *F*^2^, conventional *R*-factors *R* are based on *F*, with *F* set to zero for negative *F*^2^. The threshold expression of *F*^2^ > σ(*F*^2^) is used only for calculating *R*-factors(gt) *etc*. and is not relevant to the choice of reflections for refinement. *R*-factors based on *F*^2^ are statistically about twice as large as those based on *F*, and *R*- factors based on ALL data will be even larger.

### Fractional atomic coordinates and isotropic or equivalent isotropic displacement parameters (Å^2^)

**Table:**

	*x*	*y*	*z*	*U*_iso_*/*U*_eq_	Occ. (<1)
Mn1	0.35177 (3)	0.0000	0.27709 (5)	0.0558 (3)	
Cl1	0.40493 (4)	0.25282 (10)	0.24304 (8)	0.0815 (3)	
O1	0.5351 (5)	1.0000	0.0528 (9)	0.155 (4)	0.50
H2A	0.5257	0.9426	0.1128	0.186*	0.25
H1A	0.5745	0.9558	0.0399	0.186*	0.25
N1	0.30817 (15)	0.0000	0.4374 (3)	0.0555 (8)	
N2	0.24030 (17)	0.0000	0.2034 (3)	0.0601 (8)	
C1	0.3433 (2)	0.0000	0.5521 (4)	0.0625 (10)	
C2	0.3066 (2)	0.0000	0.6486 (4)	0.0715 (12)	
H2	0.3329	0.0000	0.7272	0.086*	
C3	0.2337 (2)	0.0000	0.6273 (4)	0.0715 (12)	
H3	0.2089	0.0000	0.6905	0.086*	
C4	0.1951 (2)	0.0000	0.5056 (3)	0.0604 (10)	
C5	0.1191 (2)	0.0000	0.4745 (4)	0.0781 (13)	
H5	0.0919	0.0000	0.5346	0.094*	
C6	0.0848 (2)	0.0000	0.3584 (4)	0.0768 (13)	
H6	0.0342	0.0000	0.3399	0.092*	
C7	0.1240 (2)	0.0000	0.2635 (4)	0.0655 (11)	
C8	0.0910 (3)	0.0000	0.1374 (4)	0.0840 (15)	
H8	0.0406	0.0000	0.1136	0.101*	
C9	0.1330 (3)	0.0000	0.0535 (4)	0.0875 (15)	
H9	0.1113	0.0000	−0.0276	0.105*	
C10	0.2073 (3)	0.0000	0.0872 (4)	0.0703 (11)	
C11	0.1985 (2)	0.0000	0.2896 (3)	0.0562 (9)	
C12	0.2350 (2)	0.0000	0.4153 (3)	0.0532 (9)	
C13	0.4239 (2)	0.0000	0.5734 (4)	0.0819 (14)	
H13A	0.4397	−0.0724	0.5150	0.123*	0.50
H13B	0.4431	−0.0434	0.6525	0.123*	0.50
H13C	0.4410	0.1158	0.5666	0.123*	0.50
C14	0.2542 (3)	0.0000	−0.0069 (4)	0.0933 (17)	
H14A	0.2713	0.1152	−0.0163	0.140*	0.50
H14B	0.2260	−0.0395	−0.0820	0.140*	0.50
H14C	0.2950	−0.0756	0.0181	0.140*	0.50

### Atomic displacement parameters (Å^2^)

**Table:**

	*U*^11^	*U*^22^	*U*^33^	*U*^12^	*U*^13^	*U*^23^
Mn1	0.0481 (4)	0.0703 (5)	0.0501 (4)	0.000	0.0127 (3)	0.000
Cl1	0.0781 (6)	0.0818 (7)	0.0876 (6)	−0.0094 (4)	0.0235 (4)	0.0054 (4)
O1	0.108 (7)	0.226 (12)	0.134 (9)	0.000	0.031 (6)	0.000
N1	0.0413 (15)	0.074 (2)	0.0503 (17)	0.000	0.0063 (13)	0.000
N2	0.0572 (17)	0.073 (2)	0.0483 (17)	0.000	0.0069 (14)	0.000
C1	0.056 (2)	0.076 (3)	0.053 (2)	0.000	0.0032 (17)	0.000
C2	0.061 (2)	0.100 (4)	0.049 (2)	0.000	−0.0002 (18)	0.000
C3	0.064 (2)	0.101 (4)	0.050 (2)	0.000	0.0127 (19)	0.000
C4	0.053 (2)	0.077 (3)	0.052 (2)	0.000	0.0100 (17)	0.000
C5	0.055 (2)	0.112 (4)	0.071 (3)	0.000	0.020 (2)	0.000
C6	0.045 (2)	0.110 (4)	0.073 (3)	0.000	0.006 (2)	0.000
C7	0.047 (2)	0.080 (3)	0.064 (3)	0.000	−0.0017 (18)	0.000
C8	0.062 (3)	0.111 (4)	0.070 (3)	0.000	−0.008 (2)	0.000
C9	0.078 (3)	0.120 (4)	0.056 (3)	0.000	−0.010 (2)	0.000
C10	0.078 (3)	0.076 (3)	0.054 (2)	0.000	0.006 (2)	0.000
C11	0.051 (2)	0.070 (3)	0.0456 (19)	0.000	0.0040 (16)	0.000
C12	0.053 (2)	0.054 (2)	0.052 (2)	0.000	0.0073 (16)	0.000
C13	0.049 (2)	0.127 (4)	0.064 (3)	0.000	0.000 (2)	0.000
C14	0.095 (3)	0.133 (5)	0.052 (2)	0.000	0.014 (2)	0.000

### Geometric parameters (Å, °)

**Table:**

Mn1—N2	2.092 (3)	C5—C6	1.347 (6)
Mn1—N1	2.140 (3)	C5—H5	0.9300
Mn1—Cl1	2.2633 (9)	C6—C7	1.421 (6)
Mn1—Cl1^i^	2.2633 (9)	C6—H6	0.9300
O1—O1^ii^	1.59 (2)	C7—C11	1.371 (5)
O1—H2A	0.8610	C7—C8	1.443 (6)
O1—H1A	0.8530	C8—C9	1.353 (7)
N1—C1	1.338 (5)	C8—H8	0.9300
N1—C12	1.345 (5)	C9—C10	1.370 (7)
N2—C10	1.342 (5)	C9—H9	0.9300
N2—C11	1.371 (5)	C10—C14	1.514 (7)
C1—C2	1.407 (6)	C11—C12	1.454 (5)
C1—C13	1.482 (6)	C13—H13A	0.9600
C2—C3	1.341 (6)	C13—H13B	0.9600
C2—H2	0.9300	C13—H13C	0.9600
C3—C4	1.428 (6)	C14—H14A	0.9600
C3—H3	0.9300	C14—H14B	0.9600
C4—C12	1.387 (5)	C14—H14C	0.9600
C4—C5	1.399 (6)		
			
N2—Mn1—N1	79.57 (12)	C7—C6—H6	119.2
N2—Mn1—Cl1	111.79 (4)	C11—C7—C6	119.7 (4)
N1—Mn1—Cl1	113.70 (4)	C11—C7—C8	115.5 (4)
N2—Mn1—Cl1^i^	111.79 (4)	C6—C7—C8	124.8 (4)
N1—Mn1—Cl1^i^	113.70 (4)	C9—C8—C7	120.4 (4)
Cl1—Mn1—Cl1^i^	119.53 (5)	C9—C8—H8	119.8
O1^ii^—O1—H2A	109.0	C7—C8—H8	119.8
O1^ii^—O1—H1A	119.0	C8—C9—C10	120.4 (4)
H2A—O1—H1A	104.5	C8—C9—H9	119.8
C1—N1—C12	117.9 (3)	C10—C9—H9	119.8
C1—N1—Mn1	129.1 (3)	N2—C10—C9	121.2 (4)
C12—N1—Mn1	113.0 (2)	N2—C10—C14	118.4 (4)
C10—N2—C11	119.1 (3)	C9—C10—C14	120.3 (4)
C10—N2—Mn1	128.4 (3)	C7—C11—N2	123.3 (3)
C11—N2—Mn1	112.5 (2)	C7—C11—C12	118.2 (3)
N1—C1—C2	122.5 (3)	N2—C11—C12	118.5 (3)
N1—C1—C13	116.6 (4)	N1—C12—C4	123.0 (3)
C2—C1—C13	121.0 (4)	N1—C12—C11	116.5 (3)
C3—C2—C1	120.0 (4)	C4—C12—C11	120.5 (3)
C3—C2—H2	120.0	C1—C13—H13A	109.5
C1—C2—H2	120.0	C1—C13—H13B	109.5
C2—C3—C4	118.5 (4)	H13A—C13—H13B	109.5
C2—C3—H3	120.8	C1—C13—H13C	109.5
C4—C3—H3	120.8	H13A—C13—H13C	109.5
C12—C4—C5	119.2 (4)	H13B—C13—H13C	109.5
C12—C4—C3	118.2 (4)	C10—C14—H14A	109.5
C5—C4—C3	122.6 (4)	C10—C14—H14B	109.5
C6—C5—C4	120.7 (4)	H14A—C14—H14B	109.5
C6—C5—H5	119.7	C10—C14—H14C	109.5
C4—C5—H5	119.7	H14A—C14—H14C	109.5
C5—C6—C7	121.7 (4)	H14B—C14—H14C	109.5
C5—C6—H6	119.2		

Symmetry codes: (i) *x*, −*y*, *z*; (ii) −*x*+1, −*y*+2, −*z*.

## References

[bb1] Bruker (2001). *SAINT-Plus*. Bruker AXS Inc., Madison, Wisconsin, USA.

[bb2] Bruker (2004). *APEX2*. Bruker AXS Inc., Madison, Wisconsin, USA.

[bb3] McCann, S., McCann, M., Casey, M. T., Jackman, M., Devereux, M. & Mckee, V. (1998). *Inorg. Chim. Acta*, **279**, 24–29.

[bb4] Pan, T.-T. & Xu, D.-J. (2005). *Acta Cryst.* E**61**, m740–m742.

[bb5] Sheldrick, G. M. (2008*a*). *SADABS*. University of Göttingen, Germany.

[bb6] Sheldrick, G. M. (2008*b*). *Acta Cryst.* A**64**, 112–122. 10.1107/S0108767307043930 18156677

[bb7] Xu, M.-L., Sun, S.-B., Li, X.-Y. & Che, G.-B. (2009). *Acta Cryst.* E**65**, m136. 10.1107/S1600536808043468 PMC2968193 21581752

